# Case report: ADHD and prognosis in tyrosinemia type 1

**DOI:** 10.3389/fpsyt.2023.1213590

**Published:** 2023-07-18

**Authors:** Helene Barone, Irene Bircow Elgen, Yngve Thomas Bliksrud, Eirik Vangsøy Hansen, Rita Rigmor Skavhellen, Magne Ivar Furevik, Jan Haavik

**Affiliations:** ^1^Regional Resource Center for Autism, ADHD and Tourette Syndrome, Division of Psychiatry, Haukeland University Hospital, Bergen, Norway; ^2^Department of Child and Adolescent Psychiatry, Division of Psychiatry, Haukeland University Hospital, Bergen, Norway; ^3^Department of Medical Biochemistry, Oslo University Hospital, Oslo, Norway; ^4^Department of Pediatrics, Haukeland University Hospital, Bergen, Norway; ^5^Bergen Center of Brain Plasticity, Division of Psychiatry, Haukeland University Hospital, Bergen, Norway; ^6^Department of Biomedicine, University of Bergen, Bergen, Norway

**Keywords:** attention-deficit hyperactivity disorder, tyrosinemia type 1, treatment compliance, neurometabolic disorders, case report

## Abstract

Neurometabolic disorders such as tyrosinemia type 1 (TYRSN1) may interfere with brain metabolism and show symptoms of attention-deficit hyperactivity disorder (ADHD) in patients treated with the enzyme inhibitor nitisinone [2-(2-nitro-4-trifluoromethylbenzoyl)-1,3-cyclohexanedione, NTBC]. It has been reported that ADHD treatment improves treatment compliance, which is imperative for the long-term prognosis of patients with TYRSN1. In this study, we report the case of a male patient who was diagnosed with TYRSN1 at 3 months of age and was subsequently treated with NTBC, restricted protein intake, and amino acids supplementation. At 7 years of age, he was referred for neuropsychiatric assessment, diagnosed with ADHD, and treated with methylphenidate. The effects of the treatment were monitored via parental interviews, questionnaires covering ADHD symptoms, and a continuous performance test. A reduction in ADHD symptoms, particularly inattentiveness, was observed across all measures. The early identification of ADHD and the treatment of neurometabolic disorders, such as TYRSN1, may be important from a lifetime perspective as this may improve the prognosis of the medical condition as well.

## Introduction

### Tyrosinemia type I

Hereditary tyrosinemia type I [(TYRSN1), OMIM 276700] is an autosomal recessive aminoacidopathy whose global incidence is estimated to be 1 in 100–120,000 live births, and its incidence in Norway is ~1 per 74,800 live births (1). TYRSN1 is caused by a deficiency in fumarylacetoacetase (FAH; E.C.3.7.1.2), the final enzyme involved in tyrosine degradation. Organ damage, including progressive liver disease with regeneration, cirrhosis, and secondary renal tubular dysfunction, occurs because the lack of functional FAH leads to the accumulation of toxic metabolites such as fumarylacetoacetate and succinylacetone. The disease's clinical symptoms widely vary as the chronic form presents with hypophosphatemic rickets during adolescence, and the most acute form presents with severe liver failure just a few weeks after birth. Without treatment, these patients die of cirrhosis or hepatocellular carcinoma (HCC) at young ages.

The treatment consists of a regimen of the drug, nitisinone [2-(2-nitro-4-trifluoromethylbenzoyl)-1,3-cyclohexanedione, NTBC], and a protein-restricted diet. The drug inhibits tyrosine degradation at an earlier step, thus avoiding the production of carcinogenic metabolites; however, the levels of P-tyrosine remain elevated or even increase. This drug dramatically improves the prognosis of TYRSN1 patients (2). The demanding diet regime aims to improve the amino acid profile in the patient's plasma to secure normal neurodevelopment. However, neurological and neuropsychological problems, including learning difficulties and symptoms of attention-deficit hyperactivity disorder (ADHD), have also been described among patients on a regimen of NTBC and dietary treatment (3–6), and treatment adherence in patients with TYRSN1 has been reported to be low (7).

### Attention-deficit hyperactivity disorder

ADHD is a neurodevelopmental disorder involving either hyperactivity/impulsivity, inattention, or both (8). It is one of the most common psychiatric disorders affecting ~5% of children worldwide (9), and its symptoms may persist in adulthood (10). Impairment of executive functioning has been described as a core deficit in ADHD, including a broad range of cognitive “top-down” processes that enable a flexible and goal-directed behavior, such as planning (11). Psychiatric conditions and learning difficulties are more common in ADHD and *vice versa*. Furthermore, several other medical conditions are associated with ADHD (12–15). The symptoms of ADHD may be present in an even higher number of diseases than previously reported, including a range of neurometabolic Mendelian disorders that affect brain development and function, some of which involve alterations in dopamine synthesis (4, 16). Research suggests that the standard pharmacological treatment (methylphenidate) could reduce the symptoms of ADHD, e.g., in phenylketonuria and TYRSN1 (5, 17), which is reason to believe that ADHD symptom reduction improves compliance to the comprehensive treatment regimen associated with (neuro)metabolic disorders such as TYRSN1 and, thereby, their prognosis (5). In this study, we examined one patient with TYRSN1 and ADHD who underwent methylphenidate treatment.

## Case report

A boy who was born healthy at birth and developed normally during the first 2 months of his life became ill with a high fever after 3 months, which led to hospitalization. He was diagnosed with *E. coli* sepsis, and elevated levels of liver enzymes, C-reactive protein (CRP), and international normalized ratio (INR) were detected. Ultrasonography and CT scans confirmed ascites, splenomegaly, and hepatomegaly with nodular structures, signs of cirrhosis, and portal hypertension. The patient was hospitalized with liver failure, and TYRSN1 was suspected. The diagnosis was confirmed by the detection of increased urinary succinylacetone levels and disease-causing mutations in the *FAH* gene. Lab results also showed low plasma albumin, low phosphate, and elevated ammonia, 112 μmol/L. Treatment was initiated with enzyme inhibitor/NTBC and dietary treatments such as restricted protein intake and adjusted amino acid supplements. During this treatment, the patient was recovered from liver failure. The plasma alpha-fetoprotein (AFP) level was initially >10,000 μg/L, but after a month, it was < 1,000, and liver enzymes also gradually normalized. The plasma AFP level was within the normal range (< 10 μg/L) after 2 years of treatment.

**Figure 1:**
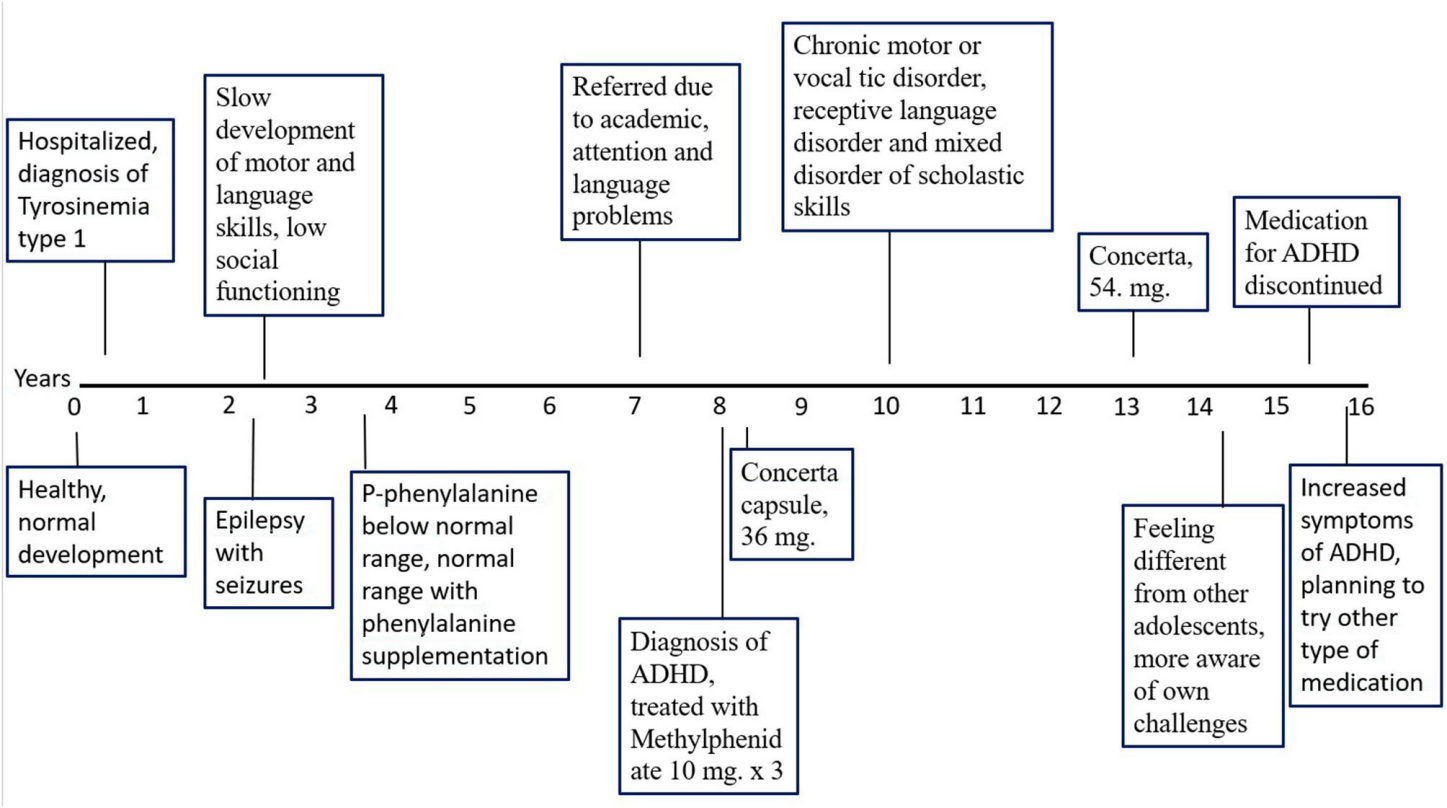
Timeline of the patients clinical course.

The patient and family managed the treatment well, and after the treatment, he showed normal physical development and plasma tyrosine levels ranging between 300 and 600 μmol/L in almost every blood test (Figure 1). The recommended plasma tyrosine concentration in T1 is 200–600 μmol/L (18). However, these treatment recommendations vary across different centers and countries, with upper tyrosine concentrations ranging between 400 and 600 μmol/L (19). At 3–5 years of age, plasma phenylalanine transiently dipped below the normal range but normalized after phenylalanine supplementation. Since the NTBC and dietary treatments were established, during check-ups, neither an indication of urinary succinylacetone nor an elevation of plasma AFP levels appeared in his samples.

After 2 years of commencing treatment, at the age of 2 years and 3 months, the patient experienced seizure episodes, initially with shaking movements in the arms and legs, blue lips, and a lack of consciousness for 5 min. The first EEG appeared normal, but the episodes recurred; Later, EEG showed possible epileptic activity. Treatment with topiramate was initiated from 3 years of age. Later, the patient developed absence seizures, following which lamotrigine was successfully administered in response. Although his epilepsy was well-controlled, his motor and language skills developed more slowly from the age of 3–5 years.

In kindergarten, he received permanent assistance because of his low social functioning. At the age of 7 years, he presented with a variety of problems, including academic challenges that required special support in school, and was referred to the Child and Adolescent Mental Health Service. He experienced problems with inattention, planning, and the organizing of activities. He also showed problems understanding information and expressing himself. Furthermore, he described himself as being anxious in new situations; he resorted to sitting under the table for ~15 min in settings with several people. In addition, he often got into conflicts with other children during school breaks.

## Methods for ADHD assessment and treatment

The diagnostic procedure for ADHD was performed according to the National Guidelines for ADHD published by the Norwegian Directorate of Health and the guidelines for ADHD treatment published by the Norwegian Medical Association. The diagnostic procedure was based on a thorough evaluation of the patient's symptoms and functional impairments.

The ADHD core symptoms (baseline and with treatment) were evaluated using the Clinician's Manual for the Assessment of Disruptive Behavior Disorders Rating Scale for Parents/Teachers (20), which is an effective rating scale for assessing attention and hyperactivity symptoms.

To assess inattention, hyperactivity, and impulsivity before and after treatment, the QB test was administered (21). This continuous performance test assesses inattention and impulsivity combined with measures of motor activity and is designed to measure the core symptoms of ADHD. The test was administered on a PC and lasted ~15 min. The participant was instructed to press a button when a circle is presented on the screen but not when there is a cross on the circle. The participant's movements were recorded using an infrared camera that follows a reflective marker attached to a headband. The hyperactivity was measured on the basis of motor activity recorded by the camera. Impulsivity and inattention were, respectively, measured through commission errors and error rate or through reaction time and omission errors. See section Supplementary material for a brief description of the measures included in the QB test.

The K-SADS-PL-2009 [Schedule for Affective Disorders and Schizophrenia for School-Aged Children (6–18 years)—Present and Lifetime (version 2009)] (22) is a semi-structured child and adolescent psychiatric diagnostic instrument used to ascertain psychiatric disorders according to the DSM-IV criteria. This was used to assess potential psychiatric comorbidities.

Guidelines for the treatment of ADHD, as edited by the Norwegian Medical Association (NMA) (23) and the Norwegian National Guidelines for ADHD, as published by the Norwegian Directorate of Health, have suggested an integrated approach to the treatment of ADHD. This involves reducing the core symptoms, improving psychosocial functions, preventing the development of additional problems, and improving functioning in everyday life. A short-acting methylphenidate formulation that lasts 3–5 h at a recommended dosage of up to 2 mg/kg was initiated for a period of 3 weeks (10 mg × 3) and evaluated.

The study was approved by the Regional Committee for Medical Research Ethics of Western Norway (IRB 00001872). Both the parents and the boy signed an informed consent form and received information about what would be presented in the article, and they were also provided the manuscript for approval before submission. All procedures were performed in accordance with the Declaration of Helsinki (of 1975, as revised in 2000).

## Results

A standard assessment of intellectual functioning was administered at the local psychological-educational service when he was 7 years of age. His intellectual functioning was below average, with the lowest score on the index measuring working memory and the highest measuring processing speed. There was no difference between verbal comprehension and perceptual reasoning. After ruling out other psychiatric disorders, it was concluded that he met the criteria for ADHD and specific phobia but no other diagnoses as per the K-SADS-PL-2009.

The effect of methylphenidate was assessed after 3 weeks of treatment. The evaluation involved (1) clinical evaluations along with the mother, (2) parents' and teacher's reports on ADHD core symptoms, and (3) changes in inattention/hyperactivity (before and after treatment) measured using the QB test.

His mother reported that their family had “got a new life.” She described him as more attentive, organized, and communicative. He also exhibited less anxiety in novel situations. In school, his learning capacity increased (reported after 6 months), and no adverse effects were observed.

Parents and the teacher reported improvements in the core symptoms of ADHD, mostly regarding attention on the Barkley ADHD questionnaire (Table 1). However, these reduced hyperactivity symptoms were reported only by the family but not by the teacher.

**Table 1:** Symptoms of ADHD in a boy with TYRSN1 before the start and after 3 weeks of treatment with methylphenidate.

**Barkley ADHD questionnaire**	**Baseline**	**Treatment**
**Parents**		
Inattention	8/9	0/9
Hyperactivity	6/9	1/9
**Teacher**		
Inattention	6/9	4/9
Hyperactivity	0/9	0/9

The results of the continuous performance test (in percentiles) for the different measures of the QB test are presented in Table 2. All but one measure showed improvements in performance. This effect was most prominent for inattention measures, especially reaction time. The highest reduction for any measure was observed for the anticipatory measure within the impulsivity scale. This means that the number of guesses (responses registered immediately before or following a stimulus) was substantially reduced.

**Table 2:** Results from the QB test in an 8-year-old boy with TYRSN1 and ADHD before and after 3 weeks of treatment with methylphenidate.

**QB test**		
**Motion**	**Non-medicated (percentile)**	**Medicated (percentile)**
Time active	96	90
Distance	92	79
Area	96	84
Microevent	95	86
Motion simplicity	88	90
**Inattention**	**Non-medicated (percentile)**	**Medicated (percentile)**
Reaction time variability	99	66
Omission error	96	66
Reaction time	96	31
Normalized variance	99	82
**Impulsivity**	**Non-medicated (percentile)**	**Medicated (percentile)**
Commission	98	92
Anticipatory	96	8
Multi-response	99	50
Error rate	98	79

### Long-term functioning and treatment

After the initial assessment, he continued with Concerta^®^ (Janssen Pharmaceutica NV, Beerse, Belgium), the proprietary name for methylphenidate, 36 mg one capsule q.a.m. and 5–10 mg of short-acting methylphenidate, on demand, in the afternoon. At approximately the age of 10 years, he developed motor tics, problems with understanding, and expressing language became more prominent, and he experienced increasing problems with academic functioning. After 3 years of the first assessment, he was also diagnosed with chronic motor or vocal tic disorder, receptive language disorder, and a mixed disorder of scholastic skills.

After 13 years of age, his symptoms of inattention increased, and the dosage of Concerta was successively increased to 54 mg q.a.m, which reduced the inattention problems. This was also when his body mass index (BMI) was in the normal range, which gradually decreased until his 15 years; thereafter, treatment with methylphenidate was discontinued at 15.5 years of age (Figure 1). As the ADHD symptoms increased after treatment discontinuation, it was planned to test other types of ADHD medications. At the age of 15 years, he still received support at school and thrived but felt different from the other pupils, being more aware of his challenges. See Figure 1 for a brief summary of the clinical course and interventions.

## Discussion

An 8-year-old boy with TYRSN1 was evaluated at the Department of Child and Adolescent Psychiatry for several neuropsychiatric symptoms, including ADHD. He was treated with methylphenidate, which had an effective response, as informed by himself, his parents, and his teacher, in addition to scores on the continuous performance test. The largest effect was observed for the symptoms of inattention.

This treatment was conducted in accordance with previous findings indicating that TYRSN1 is associated with symptoms of inattention (24) and cognitive impairment (25). We recently observed a strong correlation between the symptoms of inattention and the recent and long-term plasma tyrosine levels of patients with TYRSN1-receiving nitisinone (5). It is possible that the reported inattention and cognitive impairments are related to suboptimal metabolic control (25). In contrast to former beliefs that the high levels of plasma tyrosine found in treated patients with TYRSN1 would increase dopamine synthesis in the brain (6), it is possible that the high level of tyrosine might inhibit the production of dopamine and norepinephrine within brain tissues (5). Decreased dopamine activity in the frontal cortex is postulated to be a biological mechanism of ADHD (26) and is also found in phenylketonuria, a more frequent and better-investigated neurometabolic disorder with a high prevalence of comorbid ADHD (27, 28). Shared biological mechanisms between TYRSN1 and ADHD might also explain the increased prevalence of ADHD symptoms in patients with TYRSN1. Central stimulants, the most common treatment for ADHD, work by inhibiting the synaptic noradrenaline and dopamine transporters, thereby increasing the concentration of these catecholamines in the synapses.

Adolescents with ADHD have increased school dropout rates, substance abuse, and injuries such as motor vehicle crashes (29). Early identification and treatment, as in the present patient, may improve long-term social and educational outcomes (30). It is also a strong predictor for work participation as an adult, apparently independent of substance abuse, comorbidity, and current treatment (31). ADHD also probably affects treatment adherence in somatic diseases. For example, poor treatment adherence has been observed in adolescents with undiagnosed ADHD and type 1 diabetes (32). Nylander and Fernell (33) have emphasized the importance of screening for ADHD when type 1 diabetes is diagnosed and to perform repeated screening, especially in patients with poor metabolic control. It has been suggested that all children with neurometabolic disorders should be screened for comorbid ADHD and other neuropsychiatric disorders, especially in their first decade of life, with an in-depth assessment if the screening is positive. Adequate ADHD treatment targeting inattention and impulsivity may improve self-control and, thereby, the long-term outcomes of the neurometabolic disorder itself (4). This is supported by research suggesting that methylphenidate reduces the difficulties of executive functioning in ADHD (34) and thereby probably improves treatment compliance of the somatic condition. In the case presented here, it was observed that the stimulant treatment also improved adherence to TYRSN1 treatment.

The term “diagnostic overshadowing” has been used to describe the underdiagnosis of comorbid psychiatric conditions in severe somatic diseases. For example, one study reported that ADHD was overshadowed by neurological disorders in 71.8% of the cases (35). Interestingly, interventions targeting medical conditions can also mask ADHD symptoms. For the boy described in the present article, the teacher reported no symptoms of hyperactivity (Table 1). However, he appeared clearly hyperactive when tested despite the highly structured situation of the QB test (Table 2). Owing to his medical condition, he was permanently attended to by an assistant at school, which may explain the low rate of hyperactivity observed by his teacher. Many children with severe medical conditions may receive such support, which, paradoxically, may hinder the early identification and treatment of ADHD. In addition, psychiatric symptoms might be also explained by hospitalization and stress related to the somatic condition. Cannon Homaei et al. (4) argued that the possible underreporting of comorbid ADHD may be especially harmful to individuals with neurometabolic conditions, as they are particularly dependent on cognitive functions that are compromised by their ADHD symptoms. Furthermore, they suggested that symptoms even below the conventional threshold for a diagnosis of ADHD could cause significant problems with treatment adherence and also that patients below the diagnostic threshold may need extra help to follow the strict treatment regimen associated with these diseases.

This case also illustrates the complexity that often characterizes patients with neurodevelopmental disorders. The trajectory of diffuse problems early in life and the successive fulfillment of the criteria for different diagnoses has also been described as typical by Gillberg (36). This phenomenon highlights the importance of repeated screening and assessment of children with early neurodevelopmental symptoms, especially when somatic conditions are present.

## Strengths and limitations

This case highlights the importance of identifying the symptoms of ADHD in neurometabolic disorders and the risk of diagnostic overshadowing in such complex cases. Gathering more data on the possible relationship between ADHD and neurometabolic disorders could also provide deeper insights into the etiology of ADHD and improve the patient's clinical management (5). However, the mechanisms leading to cognitive deficits in treated TYRSN1 patients may be multifactorial; for instance, it is debated if severe liver failure in TYRSN1 leads to cognitive deficits (3). In the present case, the patient was also diagnosed with epilepsy, which may have contributed to the symptoms of ADHD.

## Conclusion

The lifestyle that sometimes accompanies untreated ADHD may be especially harmful to individuals dependent on following strict treatment regimens for their medical conditions. Early identification of ADHD and treatment of medical conditions such as TYRSN1 is important from a lifetime perspective as this may also improve the prognosis of the medical condition itself. Clinicians working both in somatic and psychiatric wards should therefore be aware of the association between ADHD symptoms in tyrosinemia type 1 and other neurometabolic conditions.

## Data availability statement

The original contributions presented in the study are included in the article/Supplementary material, further inquiries can be directed to the corresponding author.

## Ethics statement

The studies involving human participants were reviewed and approved by Regional Committee for Medical Research Ethics of Western Norway (IRB 00001872). Written informed consent to participate in this study was provided by the participants' legal guardian/next of kin. Written informed consent was obtained from the minor(s)' legal guardian/next of kin for the publication of any potentially identifiable images or data included in this article. Written informed consent was obtained from the participants/next of kin for the publication of this case report.

## Author contributions

RS and MF have made substantial contributions to the acquisition of data for the work. YB, EV, HB, and IE have made substantial contributions to the conception and design of the work, the acquisition and interpretation of data for the work, and drafting the work. JH has made substantial contributions to the interpretation of data of the work. All authors have revisited the work critically for important intellectual content, provided approval for publication of the content, and agreed to be accountable for all aspects of the work in ensuring that questions related to the accuracy or integrity of any part of the work are appropriately investigated and resolved.
