# Supplementary material for: Case report: ADHD and prognosis in tyrosinemia type 1
Source: Front Psychiatry. 2023 Jul 18;14:1213590. doi: 10.3389/fpsyt.2023.1213590 (PMC10392124; doi:10.3389/fpsyt.2023.1213590)
Supplement: Supplementary file 1 [file Data_Sheet_1.docx]

**Supplementary material**

Description of the different measures included in the QB-test

Activity

Time active: Amount of time where the participant has moved more than one centimeter in

percent of the total time of testing

Distance: How long (in meters) the marker on the headband have moved during the time of

testing

Area: Measure of the area where the movements have been placed.

Microevent: Number of times the participant has moved more than 1 mm.

Motion simplicity: Measures the complexity of movements.

Inattention

Reaction time variability: The standard deviation of the reaction time.

Omission error: Number of lacking responses despite that a signal has been shown.

Reaction time: The mean time from a Go-stimulus is shown on the screen to a correct

response has been recorded.

Normalized variance: The standard deviation divided with reaction time.

Impulsivity

Commission: Number of responses that have been recorded despite that no Go-stimulus has

been shown.

Anticipatory: If the response has been registered right before or after a stimulus has been

shown, this will be recorded as a guessing rather than a reaction to the stimulus.

Multi response: This shows how often a participant has pressed the button more than once for

each stimulus.

Error rate: This is the total sum of wrong responses to stimuli.
